# Fighting Arbovirus Transmission: Natural and Engineered Control of Vector Competence in *Aedes* Mosquitoes

**DOI:** 10.3390/insects6010236

**Published:** 2015-03-23

**Authors:** Joy Kean, Stephanie M. Rainey, Melanie McFarlane, Claire L. Donald, Esther Schnettler, Alain Kohl, Emilie Pondeville

**Affiliations:** MRC-University of Glasgow Centre for Virus Research, Glasgow, G61 1QH, Scotland, UK; E-Mails: Joy.Kean@glasgow.ac.uk (J.K.); Stephanie.Rainey@glasgow.ac.uk (S.M.R.); Melanie.McDonald@glasgow.ac.uk (M.M.); Claire.Donald@glasgow.ac.uk (C.L.D.); Esther.Schnettler@glasgow.ac.uk (E.S.)

**Keywords:** arbovirus, *Aedes*, vector control, vector competence, antiviral defences, mosquito engineering, bacteria, insect-specific viruses, paratransgenesis

## Abstract

Control of aedine mosquito vectors, either by mosquito population reduction or replacement with refractory mosquitoes, may play an essential role in the fight against arboviral diseases. In this review, we will focus on the development and application of biological approaches, both natural or engineered, to limit mosquito vector competence for arboviruses. The study of mosquito antiviral immunity has led to the identification of a number of host response mechanisms and proteins that are required to control arbovirus replication in mosquitoes, though more factors influencing vector competence are likely to be discovered. We will discuss key aspects of these pathways as targets either for selection of naturally resistant mosquito populations or for mosquito genetic manipulation. Moreover, we will consider the use of endosymbiotic bacteria such as *Wolbachia*, which in some cases have proven to be remarkably efficient in disrupting arbovirus transmission by mosquitoes, but also the use of naturally occurring insect-specific viruses that may interfere with arboviruses in mosquito vectors. Finally, we will discuss the use of paratransgenesis as well as entomopathogenic fungi, which are also proposed strategies to control vector competence.

## 1. Introduction

Female anautogenous mosquitoes need a blood meal from a vertebrate host to reproduce. Therefore, they can act as vectors for numerous pathogens, e.g., arthropod-borne viruses (arboviruses) or parasites, responsible for both human and animal diseases. The most important mosquito-borne viruses include flaviviruses (*Flaviviridae*) such as dengue virus (DENV), yellow fever virus (YFV), West Nile virus (WNV); alphaviruses (*Togaviridae*) such as chikungunya virus (CHIKV), o’nyong-nyong virus (ONNV), Semliki Forest virus (SFV), Sindbis virus (SINV); and bunyaviruses (*Bunyaviridae*) such as Rift Valley fever virus (RVFV). All of these arboviruses are mainly transmitted by aedine species including *Aedes aegypti* and *Ae. albopictus* as well as *Culex* species, with the exception of ONNV which is the only known arbovirus to be transmitted by *Anopheles*, and more specifically the African malaria vector *An. gambia*e. In recent decades, the incidence of mosquito-borne viral infections has grown dramatically. For example, according to the World Health Organization (WHO), more than 2.5 billion people—over 40% of the world’s population—are estimated to be at risk from DENV alone. A recent study has evaluated the incidence of dengue infections to be around 390 million worldwide every year, of which 96 million manifest [1]. Moreover, mosquito-borne virus infections are continuously expanding their geographical range into new areas. The spread of infections from Africa and Asia to other continents is thought to be due to extensive travelling, trade, population growth in high-risk areas, globalization of vectors, urbanization, climatic change, as well as virus genome evolution [2,3,4]. The threat of a possible outbreak of dengue fever now exists in Europe. Local transmission of DENV by *Ae. albopictus* was reported for the first time in France and Croatia in 2010, and imported cases were detected in three other European countries. Moreover, an epidemic was reported in Madeira (Portugal) in 2012–2013 in which 2200 patients tested positive for DENV, 78 of whom were known to have travelled away from the island. This spread of DENV was aided by the establishment of *Ae. aegypti* in the region. Moreover, imported cases have been detected in a further 10 European countries in addition to mainland Portugal [5,6,7,8]. In 2005–2006, an outbreak of CHIKV, normally transmitted by *Ae. aegypti*, occurred in the Indian Ocean. This epidemic has been associated with a single nucleotide mutation in the viral E1 glycoprotein conferring more efficient dissemination and transmission by the secondary vector, *Ae. albopictus*, which is more prevalent in this region [9,10,11]. Given the worldwide distribution of *Ae. albopictus*, this CHIKV variant has spread into many countries [12]. Currently, there is a large CHIKV outbreak occurring in the Americas, which started in 2013 with the first reported cases of autochthonous transmission on this continent. According to the Pan America Health Organization (PAHO)/WHO, as of February 2015, local transmission has been identified in 50 countries or territories in the Caribbean, Central America, South America, and North America. From the beginning of the epidemic in 2013 up to February 2015, a total of 1,176,216 suspected and 27,136 laboratory-confirmed chikungunya cases have been reported from these areas. CHIKV will likely continue to spread to new areas in the Americas through the movement of infected people and mosquitoes to naïve regions [4,12,13].

There are currently no vaccines for a number of important human-infecting arboviruses including DENV and CHIKV. Therefore, the control of mosquito vectors is still the main tool to eradicate, or at least reduce, the incidence of arboviral diseases [14]. Insecticides, nets and the reduction of breeding sites are currently the main tools for mosquito vector control. However, growing insecticide resistance in mosquito vectors has increasingly limited the utility of chemical insecticides [15,16,17,18]. Moreover, biting patterns of *Ae. aegypti* and *Ae. albopictus*, mainly during the day, makes the use of bed nets rather ineffective. On the other hand, larval habitats may be small, widely dispersed, and transient and it is difficult, if not impossible, to predict when and where the breeding sites will form, to find and treat them before the adults emerge. Thus, alternative methods of mosquito control are urgently needed.

The efficiency of mosquito-borne disease transmission under natural conditions is referred to as the vectorial capacity, which is modelled by the following equation, first developed for modeling malaria transmission by Ross in 1910 and later refined by others [19]:

(1) $$C=\frac{{\text{ma}}^{2}{\text{bp}}^{n}}{-\;\ln\left(p\right)}$$

This mathematical model, which can be applied to arbovirus transmission, shows that vectorial capacity (C) depends on many factors including: the abundance of vector mosquitoes per vertebrate host (m); the probability of a bite occurring (a); the probability of vector survival (p); the duration of the incubation period after which the vector can transmit the virus (n); the probability of vector survival after the incubation period is completed (1/−ln(p)); and the vector competence (b). Mosquito vector competence for arboviruses is an evaluation of the mosquito’s ability to become infected following an infectious blood meal and to subsequently transmit the virus. After the ingestion of an arbovirus by the female mosquito, it must overcome different barriers in order to be transmitted. The first is the midgut infection barrier (MIB) before the virus can invade the midgut epithelium where it undergoes replication in order to pass through the midgut escape barrier (MEB) and then disseminate to other tissues, finally reaching and infecting the salivary glands. The salivary gland infection barrier (SIB) and salivary gland escape barrier (SEB) are the final hurdles for arboviruses to overcome before they can be transmitted during a subsequent mosquito blood meal [20,21,22]. Mosquito vector competence for arboviruses has been shown to be determined by the genetic components of both the mosquito and arbovirus and their interactions, a phenomenon called genotype–genotype (GXG) interactions. Therefore, mosquito vector competence can vary among mosquito vector/arbovirus species and strains as well as between individual mosquitoes within one strain [20,23,24]. As arboviruses often exist as a collection of variable genomes within and among hosts, referred to as a mutant swarm [25], vector competence can also vary depending on the genome population of an arbovirus strain. Moreover, mosquito vector competence is dependent on environmental parameters such as temperature or symbiotic microbiota, as previously reviewed [24,26]. Thus, a considerable number of factors can influence competence, even in a same mosquito/arbovirus spp. combination.

A change in any of the parameters of vectorial capacity for a specific pathogen can affect the spread of a disease. Therefore, control of *Aedes* mosquito vectors either by mosquito population reduction or replacement with refractory mosquitoes can play an important role in the fight against arboviral infectious diseases. The reduction of populations is aimed at decreasing the probability of vector survival (p) and the abundance of vector mosquitoes per vertebrate host (m) leading to a decrease in disease transmission. Proof-of-principle experiments and field trials of such strategies in *Ae. aegypti* have already been carried out using endosymbiotic *Wolbachia* bacteria [27,28] or genetically modified (GM) mosquitoes with the RIDL (“Release of Insects carrying a Dominant Lethal”) genetic system [29]. Replacing competent mosquito populations with refractory mosquitoes aims to reduce vector competence (b). Although vector competence is not the most important determinant of vectorial capacity, vectorial capacity fluctuates directly with it as competence is a linear term in expressions of vectorial capacity. Therefore, reducing vector competence should lead to a decrease in the spread of a disease. Recently, significant advances in knowledge about the mechanisms of interactions between mosquitoes and arboviruses have been made, especially regarding the influence of mosquito antiviral responses on arbovirus propagation, genetic and phenotypic variation affecting these interactions, the impact of these interactions on mosquito fitness, and how environmental factors affect arbovirus transmission [23,26,30,31]. This opens the way for the development of novel disease control strategies by altering mosquito vector competence.

In this review, we will focus on the development and application of biological approaches, either natural or engineered, to limit mosquito vector competence for arboviruses in *Aedes* spp*.* vectors. For overall relevance, some results obtained with other mosquito species, such as *Culex* or *Anopheles*, will also be reviewed. We discuss key aspects of mosquito antiviral pathways as targets, either for the selection of naturally resistant mosquito populations or for engineering transgenic mosquitoes resistant to viral infections. We will also consider the use of endosymbiotic bacteria such as *Wolbachia*, which in some cases have proven to be remarkably efficient in disrupting arbovirus transmission by mosquitoes. Further to this, we will discuss the use of naturally occurring insect-specific viruses that may interfere with arboviruses in mosquito vectors as well as paratransgenesis of endosymbionts to control mosquito vector competence.

## 2. Natural Control of Arbovirus Transmission

### 2.1. Mosquitoes Naturally Resistant to Arbovirus Transmission

Although genetic engineering strategies to reduce *Aedes* mosquito populations [29] and the use of *Wolbachia* bacteria [32] have generated new opportunities in vector control, investigations into natural mosquito competence can serve to identify new targets for mosquito transgenic engineering or select resistant mosquitoes by breeding for population replacement strategies. As defined in the introduction, natural competence for pathogens can be due to a number of factors [24,26,30]. If environmental factors and viral properties are excluded, mosquito cellular factors such as receptors or proteins, extracellular factors and tissue-related properties (accessibility, cell types) can all potentially influence arbovirus midgut infection, replication and dissemination to the saliva. Many experimental studies have compared competence for arboviruses between regional strains of mosquito species or between species of mosquitoes and this section shall only address work where differences at genetic/molecular/physiological levels were observed that may inform vector control strategies.

One of the first observations made was that a laboratory strain of *Culex pipiens pipiens* (*Cx. pipiens pipiens*), a natural vector of WNV, was refractory to this virus. Studies on the midgut of these mosquitoes revealed an induction of apoptosis in this tissue, which the authors linked to a failure to disseminate virus from the midgut [33].

Analysis of quantitative trait loci in *Ae. aegypti* has identified targets and given further insights into the genetics of DENV competence related to midgut infection and escape barriers [34,35,36,37]. However, while these loci can be pinpointed to individual chromosomes, the host genes remain to be identified. Advances in sequencing technologies over the last three to five years may now allow these data and mosquito strains to be analyzed further and candidate regions to be assessed *in vivo*. Nonetheless, some progress on identifying the role of individual host molecules and their role in competence has been made. A putative DENV receptor present in mosquitoes that can be infected with dengue was identified [38], though more detailed descriptions and assays such as knock down studies are required to confirm this finding. More recently, Lambrechts and colleagues [39] suggested that polymorphisms in the *Ae. aegypti* Dicer2 (Dcr2, a key exogenous siRNAi pathway protein) are associated with resistance to DENV. While the exact mechanism of virus resistance due to Dcr2 polymorphisms is not known, these findings directly link mosquito innate immunity to resistance. Indeed, Dcr2 initiates the exogenous siRNA pathway by detecting and cleaving viral double-stranded RNA (induced by replication or potentially secondary structures) into 21 nucleotide virus-derived small interfering RNAs, a critical step in this antiviral response.

The establishment of new techniques for genomics and for the analysis of differential gene expression, such as microarrays and more recently high-throughput sequencing, has allowed comparative studies between refractory and susceptible mosquitoes to be performed, and are novel opportunities for in-depth genetic studies. In the case of DENV2/*Ae. aegypti* interactions, comparative studies between susceptible D2S3 and refractory Moyo-D mosquitoes have shown that common factors up-regulated in response to infection include endocytosis, autophagy regulation and a number of other physiological processes; however, some genes linked to immunity were only up-regulated in the refractory strain suggesting a further role in competence [40]. Interestingly, genes linked to one of the insect’s immune signaling pathways, the JAK/STAT pathway, previously linked to the control of DENV in this mosquito [41] were also up-regulated in both strains in response to infection. This leads to the question of potential differences in the JAK/STAT pathway between these strains (for example in expression of antiviral effectors) and whether it is really a defining contributor to competence. To analyze this question, more detailed comparative studies on this pathway between strains would be required. The same authors also compared sub-strains of the Moyo *Ae. aegypti* mosquitoes that were either resistant (MOYO-R) or susceptible (MOYO-S) to DENV2 [42]. While again confirming the important role of the JAK/STAT pathway in both strains, a number of particularly intriguing responses or genes stand out. This includes a potential role for cell death in determining the refractory state, but also up-regulation of furin-like genes which could influence the maturation of viral structural proteins in the susceptible mosquito. A transcriptome study on *Cx. pipiens quinquefasciatus* mosquitoes with different competence for WNV recently identified genes involved in ovary development as being differentially transcribed [43]. Although no further investigations were carried out, comparisons can be complicated due to differences between technical approaches, arbovirus-vector combinations as well by the microbiota environment which can affect vector competence [26,30]. Moreover, mosquito genes identified in such analyses may have potentially unknown functions. Nevertheless, all these comparative studies show that arbovirus infections can induce a strong vector response, at least by a change in the transcriptome, and this response can be different between refractory and susceptible mosquitoes. Interestingly, it has recently been shown that DENV-refractory *Ae. aegypti* mosquito strains also present higher basal levels of numerous immunity-related gene transcripts compared to susceptible strains [44]. However, silencing antiviral defence pathways does not lead to a similar increase in viral load between refractory strains. This suggests that strain-specific restriction factors could operate independently of these pathways to limit viral replication in refractory strains. Conversely, silencing negative regulators of these defence pathways in susceptible strains does not always lead to a decrease in viral titers depending on the susceptible strain and the activated defence pathway. Therefore, basal levels of the defence pathways’ transcriptome could tip the outcome of viral infection in mosquitoes. In addition, different degrees of the immune pathway activities, as well as strain-specific non-immune host factors and gene polymorphisms, contribute to control virus infection in refractory or susceptible strains [44]. Indeed, not all genes of interest may be up- or down-regulated and the identification of such candidates in the future may require other approaches, for example siRNA screens.

All these studies link differences in the vector competence phenotype to molecular and genetic factors. Naturally resistant mosquitoes could thus be selected in the laboratory for replacement strategies. However, potentially many genome loci and gene products can affect mosquito competence for arboviruses. Thus, more studies will be required not only to understand the effect of genotypic variation and their interactions on controlling vector competence but also the influence of the environment on specific vector competence genotypes [24]. Moreover, mass rearing of naturally resistant populations from a few selected individuals to obtain refractory populations still needs to be carefully assessed. Indeed, the causes of variation resulting in different competence phenotypes between individual mosquitoes within one population is a challenge that has barely been explored [24]. A reduced genetic background in the founder population could also lead to an inbreeding depression after many generations [45]. With the exception of the selection of mosquitoes that are naturally resistant to arboviruses, these studies could provide important information for genetically engineering refractory mosquitoes. However, many of the potential targets identified in these screens will have to be assessed for their function and role in competence by knock down studies or other genetic approaches. This is becoming increasingly feasible as interest in vector biology is growing and more facilities for this work are being built or upgraded. Moreover, advances in engineering mosquitoes, as outlined elsewhere in this review, are hoped to be powerful enough in the coming years to implement the information gained from these interactions and host response studies in practice. Similar studies with alphaviruses and bunyaviruses remain to be carried out but the examples of DENV and WNV show that these are potentially very informative routes of investigation.

### 2.2. Insect-Specific Endosymbionts to Decrease Mosquito Competence for Arboviruses

#### 2.2.1. Bacteria and Their Influence on Arbovirus Transmission: The Case of *Wolbachia*

**Figure 1:**
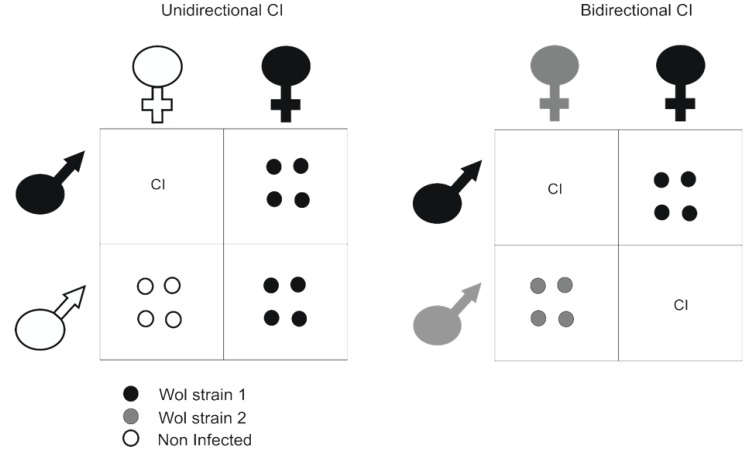
*Wolbachia* induced cytoplasmic incompatibility (CI) by infection. *Wolbachia* are spread and maintained in *Aedes* populations through a process known as CI which can be present in two distinct forms. Unidirectional CI involves infected females being able to successfully mate with both uninfected males in addition to those infected with the same or similar strains of *Wolbachia*. Bidirectional CI occurs in males and females infected with two different strains of *Wolbachia* which are unable to produce viable offspring. Both forms of CI result in females infected with *Wolbachia* having a fitness advantage. Circles indicate viable offspring and are color coded to demonstrate infection status. CI indicates where no viable offspring are produced.

According to meta-analyses, an estimated 66% of arthropod species are thought to be infected with the intracellular α-proteobacterium *Wolbachia* [46,47]. *Wolbachia* spp*.* have been extensively studied due to their symbiotic relationship with the host, often leading to significant biological changes [48]. Critical to *Wolbachia*’s successful invasion of an arthropod species is their ability to manipulate host reproduction. In populations of mosquitoes and other arthropods, *Wolbachia* are spread and maintained through a phenomenon known as cytoplasmic incompatibility (CI) [48], which can be either unidirectional or bidirectional (Figure 1) [49]. Bidirectional CI results when males and females in a given population are infected with different strains of *Wolbachia* and viable offspring can only result from the mating of males and females infected with the same strain of *Wolbachia*. Unidirectional CI occurs when only one strain of *Wolbachia* is present in a population. In this situation, uninfected females only produce viable offspring with uninfected males, in contrast to infected females that are able to produce viable offspring with both infected and uninfected males; thus, giving these infected females a fitness advantage over uninfected females. This phenomenon offers an intriguing possibility for the control of vector populations. As early as the 1960s, release experiments with mosquitoes infected with *Wolbachia* were carried out in order to eliminate wild *Cx. pipiens* populations [50]. In recent times, male *Aedes polynesiensis* mosquitoes transinfected with *Wolbachia* were also released into wild populations in order to limit the population number through CI [51].

Despite the wide occurrence of *Wolbachia* within insect species, there are no accounts of natural infections occurring in the important arbovirus vector *Ae. aegypti*. However, *Ae. albopictus* is known to be infected with at least two strains of *Wolbachia* [52,53,54]. Other mosquitoes, such as *Cx. quinquefasciatus* and *An. gambiae*, have been shown to be naturally infected with *Wolbachia* [55,56]. Before a recent study in *Anopheles*, it was assumed that like *Ae. aegypti*, *An. gambiae* were not naturally infected with *Wolbachia* [56]. This poses the question of whether we have simply not found wild populations of *Ae. aegypti* infected with *Wolbachia*. Indeed, if these populations do exist, they may have interesting characteristics with regards to arbovirus transmission that could be exploited. Apart from natural *Wolbachia* infections, studies have indicated that *Ae. aegypti* and *Ae. albopictus* can be stably transinfected with *Drosophila*-derived strains of *Wolbachia* [27,57,58]. A virulent laboratory strain of *Wolbachia* originally isolated from *Drosophila*, called *wMelPop*, was found to be life shortening when transinfected into *Aedes* populations; although, it was stably maintained [57,59,60]. The life shortening phenotype of *wMelPop* was considered to be a viable mechanism for limiting transmission of arboviruses. Indeed, female mosquitoes can take several blood meals throughout adulthood, and shortening this period would potentially result in less arbovirus transmission.

The full potential for *Wolbachia* as a tool to limit arbovirus transmission by mosquitoes became clear in 2008 when two independent studies showed that a *Wolbachia* infection in *D. melanogaster* led to a decrease in the replication and titers of several RNA viruses [61,62]. Subsequent studies have since indicated that *Wolbachia* transinfection can inhibit arbovirus transmission depending on the *Wolbachia*-mosquito-arbovirus combinations [55,61,63,64,65,66,67,68].

In *Ae. albopictus* infected with natural strains of *Wolbachia*, the presence of the endosymbiont does not lead to viral resistance in these mosquitoes and at best gives limited protection. For example, Bian *et al.* showed that the presence in *Ae. albopictus* of the native *Wolbachia* strains *wAlbB* and *wAlbA* did not significantly inhibit DENV replication [63]. However, further studies indicated that dissemination to the salivary glands was reduced, suggesting that this *Wolbachia*-host combination may limit DENV transmission [69]. Further to this, one study has indicated that *wAlbB* and *wAlbA* infected *Ae. albopictus* may, to some extent, be able to control the amount of CHIKV replication; however, not to a level that would allow the control of viral dissemination through the mosquito [70].

The mechanism(s) underlying the inhibitory phenotype are not well understood. Several key studies have indicated the importance of *Wolbachia* density in order to elicit an antiviral response or effect. For example, native *Wolbachia* are often present in high densities in *Drosophila* infections and as such offer natural viral protection [71]. Similarly, the transinfection of non-native *Wolbachia* often leads to a high density of the bacteria which has been linked to an antiviral effect [72]. A possible mechanism for the inability of native *Ae. albopictus* *Wolbachia* to efficiently control arbovirus infection in this species is that bacteria are present at very low densities [71,72]. This could suggest that a shared evolution history between *Aedes* mosquitoes/arboviruses and *Wolbachia* may have led to a reduced density of *Wolbachia*. Nonetheless, even in absence of a complete understanding of the inhibitory mechanisms, this strategy is being pursued. Artificial transinfections of *Drosophila-*derived *Wolbachia* strains into *Aedes* populations, most often *Ae. aegypti* can produce viable mosquitoes. Stable *wMelPop* transinfected *Ae. aegypti* were initially generated by firstly adapting *wMelPop* to an *Ae. albopictus*-derived cell line [73] prior to being transferred into an *Ae. aegypti* cell line for several passages before transinfection into *Ae. aegypti* mosquitoes [57]*.* Not only does this induce a life shortening phenotype and CI in this mosquito species, but several studies have also shown reduced proliferation of arboviruses, including DENV, CHIKV, YFV and WNV [66,67,68]. In addition, the introduction of another *D. melanogaster* derived strain, *wMel,* into *Ae. aegypti* does not limit the vector’s lifespan but does induce CI and is able to inhibit DENV [58,63], CHIKV and YFV [67] but interestingly not WNV [66]. Transinfection of *wAlbB* into *Ae. aegypti* is also able to inhibit DENV [63]. Similarly, *Ae. albopictus* has been transinfected with *wMel* again resulting in lower viral titers upon CHIKV infection [65].

Although the possibilities associated with *Wolbachia* are becoming increasingly recognized, the underlying mechanisms that block arbovirus infections are not as it is still unclear exactly how *Wolbachia* control arbovirus replication or transmission, with the exception of the density-based hypothesis described above [26,32,74,75]. It is known that upon transinfection of *Wolbachia*, there is an up-regulation of some innate immune pathways in *Ae. aegypti* [76,77]. It has therefore been suggested that *Wolbachia* may offer antiviral protection by “priming” the immune system against incoming arboviral infections. In contrast, immune priming is not seen in naturally occurring *Wolbachia* infections, at least in *Drosophila* where viral protection is still seen [61,62,78,79]. Therefore, immune priming may not, or at least not always, be required for *Wolbachia*’s antiviral activity, even if it could enhance the bacteria’s viral interference [76]. Other hypotheses to explain the antiviral effect conferred by *Wolbachia* have been proposed, including tissue tropism and competition for host cell resources [26,32].

Nonetheless, the real test of *Wolbachia*-mediated strategies lies in release experiments. Several of these experiments have been carried out in order to assess the success of the introduction of *Wolbachia* into wild populations. Initial studies are encouraging and show that transinfected *Ae. aegypti* are able to effectively invade a small area/population of wild mosquitoes and produce a high infection rate [27,80]. Interestingly, a recent study in Australia indicated that *Wolbachia* infection was sustained a year following field release [81]. Upon experimental infection of mosquitoes collected from the transinfected population, DENV dissemination was shown to be reduced indicating that there is a persistent antiviral effect [81]. However, it is important to note that the release area did not have an ongoing DENV outbreak and any possible effect of a shared history between *Wolbachia* and DENV on antiviral protection could not be determined. The release of *Wolbachia* into wild populations requires extensive knowledge of the local and regional mosquito population, if fixation (*i.e.*, stable introduction of *Wolbachia* into a previously naïve population) is to be successfully obtained [82,83]. Calculations of parameters such as the strength of CI, maternal inheritance rate and fitness cost of *Wolbachia* infection/transinfection must be performed [84]. Modelling experiments are then carried out to determine the minimum number of *Wolbachia* infected mosquitoes to be released [58,80,85,86,87]. These parameters are influenced by the presence of an existing *Wolbachia* infection in wild populations. Thus, local wild populations should be tested for the presence of *Wolbachia* prior to release; although, this may not be required for *Ae. aegypti*. Additionally, a recent study in *Anopheles* indicates that the natural gut flora of mosquitoes is able to inhibit the vertical transmission of *Wolbachia* [88]. Given that populations of mosquitoes in the wild are likely to vary in the composition of their gut flora, the spread of *Wolbachia* in these populations may be affected. Finally, it is also conceivable that the introduction of *Wolbachia* into wild populations may have an effect on the endogenous viruses present in these populations (see Section 2.2.2 on insect-specific viruses below) and it is unclear how this may impact on the ability to transmit arboviruses in the long term.

#### 2.2.2. Insect-Specific Viruses (ISVs) and Their Influence on Arbovirus Transmission

In recent years, an increase in the systematic virus discovery in mosquitoes and other arthropods around the world has resulted in the documentation of insect-specific viruses (ISVs). These viruses are often characterized by their specificity to replicate in certain insects—for example mosquitoes—and inability to replicate in vertebrate (cells). The first viruses discovered in this group belong to the *Flavivirus* genus (family *Flaviviridae*), with cell fusing agent virus, Kamiti River virus and Culex flavivirus (CxFV) as well-known members of these insect-specific flaviviruses (ISFs) [89]. In recent times, mosquito-specific viruses belonging to other families/genera (*Togaviridae*, *Bunyaviridae*, *Densovirinae* and *Mesoniviridae*), or as yet unclassified, have also been identified [90]. These data highlight the fact that a great number of mosquito strains in the wild are infected with mosquito-specific viruses. Although little is known about the transmission of these viruses, experimental data present for some of the ISVs as well as other supporting evidence, including the widespread nature of ISVs in wild mosquitoes, suggests that vertical transmission is the main factor for propagation [91,92,93,94]. Moreover, several well established mosquito cell cultures made from embryonic or larval mosquitoes have been reported to be persistently infected with ISVs [92,95,96]. Artificial ISV infections in naïve mosquitoes and cell lines often result in pathogenicity; however, the survivors show non-pathogenic persistent infection; although they still actively produce virus which can be transmitted to the offspring in a non-pathogenic matter [92,96,97,98]. Therefore, it can be expected that a wide number of mosquitoes are already persistently infected with ISVs at the time they encounter arboviral infections, resulting in mosquitoes infected with at least two different viruses (ISV and arbovirus) [99].

Little is known about the influence of these ISVs on arboviral infections in mosquitoes (Table 1). For several viruses (at least those belonging to *Flaviviridae* and *Togaviridae*), it has been reported that sequential infections (*i.e.*, superinfection) by the same virus, or phylogenetically closely related viruses, in the same cells are inhibited, known as superinfection exclusion [97,100,101]; however, the mechanisms underlying this process are often not known. Some ISVs—for example, insect-specific flaviviruses—are phylogenetically classified in the same genus as several arboviruses that can be transmitted from mosquitoes to vertebrates. This is in contrast to others that do not have a counterpart that is able to be transmitted from invertebrates to vertebrates—for example, densoviruses (DNVs, small non-enveloped single stranded DNA viruses; *Parvoviridae* family, ambidensovirus genus). Therefore, it could be expected that superinfection exclusion happens regularly in nature and could possibly be used as a self-sustaining control measure. It is currently not known if ISVs and arboviruses share enough phylogenetic similarity for superinfection exclusion to occur; and even if theoretically possible, if this really happens in nature. However, ISVs could also trigger immune pathways in the female mosquito and thereby potentially prime the mosquito in a superinfection exclusion unspecific way, thus still limiting further infection by arboviruses not phylogenetically closely related to ISVs.

Most current research has focused on dual infections of ISFs and mosquito-borne flaviviruses, more specifically CxFv and WNV either in *Ae. albopictus*-derived C6/36 cells or *Culex* mosquitoes [91,98]. Some reported an inhibitory effect, either by decreased viral titers or delayed disseminations, while others observed no effect. Recently, interference of a mosquito-borne flavivirus by two additional ISFs has been reported *in vitro*, resulting in lower titers of the mosquito-borne flavivirus [97,102]. Moreover, one of them showed that the inhibitory effect of the insect-specific flavivirus, Palm Creek virus, was specific to sequential infection by mosquito-borne flaviviruses (WNV and Murray Valley encephalitis) and could not be observed during the sequential infection of a mosquito-borne alphavirus (Ross River virus) [97]; this would support the superinfection exclusion theory. On the other hand, the inhibitory effect or increased pathogenicity of the mosquito-borne flavivirus DENV in mosquito cells or mosquitoes persistently infected with the ambidensovirus DNVs [96,103], supports the ability of ISVs to interfere with arboviral infections even if they belong to different virus families within some combinations.

**Table 1:** Mosquito dual infections with insect-specific viruses (ISVs) and mosquito-borne viruses and their consequence on viral infection.

Arbovirus	ISV	Experimental Host	Experimental Outline	Effect on ISV	Effect on Arbovirus	Reference
DENV2 (flavivirus)	DNV (ambi-densovirus)	*Ae. albopictus* (adults & larvae)	DNV persistent infected mosquitoes, followed by DENV2 infection	Titer increase 2–3 log	100× lower titer	[103]
WNV (flavivirus) MVEV (flavivirus), RRV (alphavirus)	PCV (flavivirus)	*Ae. albopictus* C6/36	Arbovirus 6–7 dp PCV infection		Lower titer for WNV and MVEV No effect on RRV	[97]
WNV (flavivirus)	CxFv (flavivirus)	*Ae. albopictus* C6/36	Arbovirus 48 hp CxFV infection		1 log lower titer at 108 hp infection. Other time points no effect	[91]
WNV (flavivirus)	CxFv (flavivirus)	*Cx. quinquefasciatus*	CxFv persistent infected mosquitoes, followed by WNV infection		Delay in dissemination	[91]
DENV2 (flavivirus)	*Aal*DNV (ambi-densovirus)	*Ae. albopictus* C6/36	Acute *Aal*DNV infection, followed by DENV2 infection Persistent *Aal*DNV infection, followed by DENV2 infection		Increased CPE Decreased CPE	[96]
WNV (flavivirus)	CxFv-Izabal (flavivirus)	*Ae. albopictus* C6/36	WNV 48 hp CxFV infection		Lower WNV titer from 4 dp infection (not significant)	[98]
WNV (flavivirus)	CxFv-Izabal (flavivirus)	*Cx. quinquefasciatus* Honduras/Sebring	Co-infected by injection	CxFv in salivary glands (Honduras *Cx.quinquefasciatus*) if co-infected with WNV only	Increased WNV transmission	[98]
WNV (flavivirus) JEV (flavivirus) SLEV (flavivirus)	NHUV (flavivirus)	*Ae. albopictus* C6/36	Arbovirus post or co-infected with NHUV		Lower titers	[102]

DENV, dengue virus (*Flaviviridae*, flavivirus); DNV, densovirus (*Parvoviridae*, ambidensovirus); WNV, West Nile virus (*Flaviviridae*, flavivirus); MVEV, Murray Valley encephalitis virus (*Flaviviridae*, flavivirus); RRV, Ross River virus (*Togaviridae*, alphavirus); PCV, Palm Creek virus (*Flaviviridae*, flavivirus); CxFv, Culex Flavivirus (*Flaviviridae*, flavivirus); *Aal*DNV, *Aedes albopictus* densovirus (*Parvoviridae*, ambidensovirus); JEV, Japanese encephalitis virus (*Flaviviridae*, flavivirus); SLEV, Saint Louis encephalitis virus (*Flaviviridae*, flavivirus); NHUV, Nhumirim virus (*Flaviviridae*, flavivirus); hp, hours post; dp, days post; CPE, cytopathic effect.

Even if most experimental set ups to date have focused on the inhibitory effects on the arbovirus infection in the case of persistent ISV infections, representing the natural infection order, some experimental results suggest that the order and timing of the infections can affect the interaction and therefore the effect on the arbovirus infection outcome [96,98,102]. For example, after sequential infection with DENV, *Ae. albopictus* (*Aal*)DNV acutely infected C6/36 cells show increased cytopathic effects (CPE), while *Aal*DNV persistently infected C6/36 cells show decreased CPE [96].

The large genetic diversity between ISVs, mosquito-borne viruses and mosquitoes suggest that the influence of ISVs on arboviral vector competence may indeed vary depending on the mosquito-ISV-arbovirus combination [89,90]. This is supported by Kent *et al.* who reported an effect on WNV transmission in a colony of *Cx. quinquefasciatus* from Honduras if co-injected with an ISF from Guatemala (CxFV-Izabal) which was not found in a different *Cx. quinquefasciatus* colony (Sebring). Interestingly, in contrast to the inhibitory effects other studies show, they report an increase in WNV transmission [98]. This is in line with a reported ecological association between CxFV and WNV in field collected mosquitoes whereby *Cx. pipiens* positive for WNV were more likely to be positive for CxFV [99]. However, the increase in WNV transmission reported by Kent *et al.*, in contrast to the other studies, could also be due to differences of virus administration (blood feeding or injection). On the other hand, Bolling *et al.* have reported an early suppression of WNV in CxFv persistently infected *Cx. pipiens*, resulting in delayed transmission of WNV [91].

These examples highlight the fact that although ISVs have a potential to be used to control arbovirus infections, more research is required to understand these interactions in detail before they could potentially be used as biological control measures.

### 2.3. Entomopathogenic Fungi and Their Effect on Competence for Arboviruses

To control adult mosquito vectors, a further alternative biological approach is the use of entomopathogenic fungi such as *Metarhizium anisopliae* (*M. anisopliae*) and *Beauveria bassiana* (*B. bassiana*). For a decade, these entomopathogenic fungi have been considered to be good candidates for decreasing pathogen transmission by mosquitoes mainly because of their ability to shorten the mosquitoes’ life span or reduce the blood feeding success of female mosquitoes, both in *Anopheles* species malaria vectors [104,105,106,107] (further reviewed in [108]), and in arbovirus vectors such as *Ae. aegypti* and *Ae. albopictus* [109,110,111].

In addition, when *Anopheles* or *Aedes* adult mosquitoes treated with fungi take an infected blood meal, they are less competent for transmitting the pathogen to a suitable vertebrate host [104,112,113]. In *Ae. aegypti*, both *B. bassiana* and *M. anisopliae* reduce vector competence for DENV2 [112,113]. Interestingly, it has been shown that the Toll pathway, an insect immune signaling pathway, is activated in response to fungal infections in *Ae. aegypti*. This results in the transcriptional activation of several antimicrobial peptides (*i.e.*, Cecropin, Defensin, Attacin, Diptericin and Serpin) and other immune genes [112,114]. Similarly, another immune pathway, the JAK/STAT pathway, responds to fungal infections in *Ae. aegypti* by up-regulating the transcription factor of JAK/STAT, Dome, and the dengue virus restriction factor, DVRF [112]. Studies have indicated that when Rel1 (Toll pathway controller), Dome or DVRF-1 (JAK/STAT pathway) are knocked down, *Ae. aegypti* adult females are more susceptible to fungal infections showing that Toll and JAK/STAT immune pathways exert an anti-fungal immune defence in the mosquito [112,114]. As the Toll and JAK/STAT pathways have been identified as being antiviral against DENV infection in female *Ae. aegypti* mosquitoes [41,112,115], the fungi are suspected to inhibit DENV infection at least through the activation of the Toll and JAK/STAT pathways [112].

As entomopathogenic fungi decrease mosquito vector longevity, blood feeding success and viral infection in females (therefore reducing the number of mosquitoes that can bite and get infected), entomopathogenic fungi could be used as a biological tool to control arboviral transmission by mosquito vectors and therefore limit mosquito-borne diseases [108]. Both *M. anisopliae* and *B.* *bassiana* have been shown to decrease *Aedes* vectors’ survival and feeding success in semi-field conditions [110,111]. However, before the wider use and application of fungi in the field, the way different fungal strains interact with arboviruses in mosquitoes needs to be addressed to demonstrate their potential for reducing disease transmission. In addition, it should be determined whether an eventual selection of genetic resistance to fungi could have collateral consequences for mosquito competence [116]. Besides, long-lasting formulations and optimization of delivery methods will be required to achieve efficient vector control. To date, few trials have evaluated the use and delivery methods of fungi and their impact on mosquito vectors and disease transmission in the field. So far, results indicate that the delivery methods should exploit natural behavior of mosquitoes and be used as part of an integrated vector management strategy to be an efficient control tool [117,118,119]. Finally, if entomopathogenic fungi have a large host-range of target insects, then selected fungi, as well as delivery methods, should be evaluated with caution to minimise the risk of contaminating non-target insects further to assessing their safety regarding vertebrates and environment [108,120].

## 3. Engineered Control of Mosquito Arboviral Competence

### 3.1. Engineering Mosquitoes with an Altered Arboviral Competence

The recent availability of *Anopheles*, *Aedes* and *Culex* mosquito genomes [121,122,123,124,125] in addition to progress made in molecular biology, genomics and functional genomics together with the development of mosquito transgenic methodologies, have opened the way for the generation of novel vector control strategies using genetically engineered mosquitoes either to suppress or replace vector populations. While population suppression aims to reduce the number of disease-transmitting mosquitoes, population replacement aims to substitute wild type mosquitoes with ones that have impaired vector competence in order to block pathogen transmission [29,126,127,128]. Recent significant advances in our understanding of mosquito–arbovirus interactions and the mechanisms of how *Aedes* and *Culex* mosquito vectors mount a response to control arbovirus replication and infection [30,129,130,131] have provided new insights into the molecular bases of vector competence, which should help to engineer GM refractory mosquitoes that do not transmit arboviruses [31]. Here, we discuss potential targets and focus on mosquito genes and molecular responses that have been functionally characterized as influencing mosquito vector competence for arboviruses.

#### 3.1.1. Engineering Mosquitoes with Enhanced Antiviral Defences

Among the antiviral defence pathways in mosquito vectors, the sequence specific RNA break-down mechanism RNA interference (RNAi) is a key player in limiting arbovirus replication [30,130,132,133]. RNAi can be divided into several pathways depending on the small RNAs involved: small interfering (si)RNA, micro (mi)RNA and Piwi interacting (pi)RNAs. siRNAs or virus-derived siRNAs (viRNAs) are involved in the first antiviral RNAi pathway reported in mosquitoes: the exogenous siRNA pathway, which is an important pathway targeting arbovirus infections. The siRNA/viRNA pathway is induced by long double stranded (ds)RNA, which in the case of RNA viruses, could be viral replication intermediates, secondary structures in the viral RNA or virus genomes (in the case of dsRNA viruses). The dsRNA is recognized by the enzyme Dcr2 and cut into 21 nt long siRNAs/viRNAs, which in turn are taken up by the RNA induced silencing complex (RISC) harboring Argonaute 2 (Ago2) as the catalytic component. The siRNAs/viRNAs are then unwound and one strand is kept as a guide to find complementary (viral) sequences resulting in slicing and degradation of the specific (viral) RNA [130,133]. Transient knock down of the key proteins, Dcr2 or Ago2, in whole mosquitoes and derived cell lines, mainly from *Aedes*, results in an increase in replication of a variety of arboviruses from different families (DENV, CHIKV, SFV, ONNV, SINV) [134,135,136,137,138,139]. Nevertheless, little is known about the *in vivo* tissue-specificity of the siRNA/viRNA pathway, and it is still not clear in which mosquito tissues this pathway is endogenously expressed and active to protect the mosquito from arboviral infections. In *Ae. aegypti*, the exogenous siRNA pathway appears to be active and modulate vector competence for arboviruses at least in the midgut. Indeed, transgene-mediated silencing of Dcr2 in the midgut of *Ae. aegypti* leads to significantly higher SINV titers in this tissue and dissemination rates of the virus throughout the rest of the mosquito [140]. Similarly, when a suppressor of the siRNA pathway, the Flock house virus B2 protein (FHV-B2), is overexpressed in the midgut of transgenic *Ae. aegypti*, SINV titers in the midgut and dissemination rates increase [141]. This further indicates that the SINV strain used in these two latter studies encounters a dose-dependent MEB in *Ae. aegypti*, and that the siRNA pathway controls this virus barrier in this instance. When the same transgenic mosquitoes are infected with DENV, the midgut infection titers increase, although to a lesser extent compared to SINV, indicating that the siRNA pathway also controls DENV replication in the midgut. However, in contrast to SINV infection, this does not lead to higher dissemination rates. It is possible, as mentioned by the authors of the study, that DENV failed to overcome a dose-dependent MEB because the viral titers in the midgut are still low and that the siRNA pathway is not the main factor controlling the dose-dependent MEB for this virus. However, the American DENV strain used in this study is known to be poorly efficient in overcoming the MEB in *Ae. aegypti* compared to other DENV strains [142,143,144]. Therefore, it is also possible that even if the siRNA pathway and others factors are capable of limiting DENV titers in the midgut cells, this particular virus strain may be confronted by a dose-independent MEB, such as an incompatibility between *Ae. aegypti* and this arbovirus strain, preventing the virus from exiting the midgut epithelial cells [141]. In contrast to *Ae. aegypti*, it has been recently shown in the malaria mosquito *An. gambiae* that the siRNA pathway does not influence ONNV midgut infection but only reduces virus replication in the systemic compartment after virus dissemination from the midgut [145]. Interestingly, the siRNA pathway is nevertheless active and functional in the midgut after ONNV infection, yielding viRNAs derived from the ONNV genome. This latter result suggests that this pathway could play a role in virus genome “antigen”-detection and immune system signaling rather than as a direct killing agent before viral dissemination from the midgut [145].

Recently, in addition to viRNAs, arbovirus-specific piRNAs have been detected in *Ae. aegypti* and *Ae. albopictus* mosquitoes and their derived cell lines for arboviruses belonging to different families (*Togaviridae*: SINV, SFV and CHIKV; *Flaviviridae*: DENV; *Bunyaviridae*: RVFV, Schmallenberg virus and La Crosse virus), suggesting that the piRNA pathway also displays antiviral activity [138,146,147,148,149,150]. Little is known about the piRNA pathway in arthropods and even less in mosquitoes. Most of our understanding comes from the model organism *D. melanogaster*. Here, piRNAs are known to be important for genome stability by targeting transposons in the germline and surrounding cells. piRNA molecules harbor specific features due to their unique production pathways. The piRNA pathway can be divided into two parts. The first part results only in primary piRNAs (antisense with a bias for Uridine at position 1) and involves the Piwi and Aubergine proteins. This pathway is present in both somatic and germ line cells. The second part of the pathway involves an additional ping-pong amplification of the piRNAs, resulting in primary and secondary piRNAs (sense, with a bias for Adenine at position 10). This amplification loop involves the Ago3 protein and is, at least in the fruit fly, restricted to germline cells [133,151]. In contrast to *D. melanogaster* which has one PIWI, one Aubergine and one Ago3 protein, *Ae. aegypti* has seven PIWI, no Aubergine and one Ago3 protein [152]. Moreover, transcripts of Piwi 2–7 and Ago3 can be found in somatic tissue of *Ae. aegypti* [138,146,148], suggesting some differences between the piRNA pathway in the fruit fly and *Ae. aegypti*. Combined knock down experiments of all Piwis and Ago3 in *Ae. aegypti* derived Aag2 cells followed by arbovirus (SFV) infection, suggested the involvement of these proteins in the production of viral specific piRNAs. Moreover, specific knock down of Piwi4 resulted in an increase of SFV infection [138]. Similarly, depleting Ago3 in *An. gambiae* mosquitoes increased ONNV titers [134], supporting an antiviral function of the piRNA pathway at least in mosquitoes.

miRNAs are small RNA molecules shared by many organisms to post transcriptionally regulate transcripts. miRNAs mainly down-regulate transcript expression by translational inhibition or RNA degradation; although, positive regulation has also been reported. The miRNA pathway can be divided into canonical and non-canonical pathways and although it shares several similarities with the siRNA pathway, it encodes for specific miRNA pathway effector proteins. In contrast to the siRNA pathway which is solely cytoplasmic, miRNAs have both a nuclear and cytoplasmic phase. In the canonical pathway, transcripts from miRNA clusters are transcribed and folded back into partial dsRNA stem-loop structures called primary (pri-)miRNAs. Pri-miRNAs are cleaved into shorter precursor (pre-) miRNAs by Drosha, followed by export of the pre-miRNAs from the nucleus into the cytoplasm. It is here that Dcr1 cuts these into 21/22 nt miRNA/miRNA * duplexes which in turn are taken up by the miRISC harboring Ago1 as the catalytic compound. After unwinding the duplex, one strand is used as a guide to find (partial) complementary RNAs, resulting in their degradation or translational inhibition. Also, in contrast to siRNAs, miRNA/miRNA* duplexes are often not perfect dsRNA duplexes and can act on partially complementary target RNAs [133,153]. The non-canonical pathway shares many of the features with the canonical pathway; however, pre-miRNAs can also be produced independently from Drosha due to splicing and some miRNAs have been reported to be Ago2 dependent rather than Ago1 [154,155,156,157]. Evidence that the miRNA pathway in mammals acts antivirally is increasing. This can be either due to direct targeting of the viral RNA or indirect targeting by changing the environment in the cell to be less or more favorable for virus infection [158]. In mosquitoes, the transient knock down of the miRNA effector proteins Ago1 and Dcr1 did not result in up-regulation of replication for several mosquito-borne viruses (like alphaviruses), in contrast to the knock down of the exogenous siRNA effector proteins (Ago2 and Dcr2) [134,138,139]. However, changes in the miRNA profile in infected *versus* non-infected mosquitoes or their derived cell lines have been reported for WNV (*C. quinquefasciatus* mosquitoes and *Ae. albopictus* C6/36 cells) [159,160], DENV (*Ae. aegypti* mosquitoes and C6/36 cells) [161,162] and CHIKV (*Ae. albopictus* mosquitoes) [163]. For most of the identified mosquito miRNAs, it is not yet known how important they are for arbovirus infection in mosquitoes and what their targets are. In addition, WNV and DENV have been reported to encode miRNAs in their viral genome, which can either target vector or viral transcripts [164,165].

Thus, genetically enhancing RNAi pathways in mosquitoes could reduce their competence for arboviruses and may provide a powerful tool for replacement strategies [128,130]. Several proteins involved in the RNAi response could also be used as potential targets for genetic control strategies; however, some are yet to be fully characterized to ensure their effect is limited to arbovirus infection and to minimize the risk of unwanted side effects. The exogenous siRNA pathway and its effector proteins (Dcr2 and Ago2) are potential targets that have broad antiviral activity and are currently the best studied. Proteins of the piRNA pathway also have the potential to be targets of genetic control strategies; however, more research is needed to investigate their involvement in the antiviral response against other arboviruses and their importance in mosquito genome stability. Targeting effector proteins of the miRNA pathway has a high chance of causing undesirable side effects in mosquitoes due to the importance of this pathway in gene regulation. Instead of targeting proteins of the RNAi pathway itself, some small RNA pathways can also be used directly. As a proof-of-principle, transgenic lines of *Ae. aegypti* which express a DENV2 sequence-derived dsRNA (following transcription of inverted-repeats), in the midgut or salivary glands, have been successfully used to artificially trigger the siRNA pathway against the DENV and exhibited reduced vector competence for DENV2 but not for other DENV serotypes [166,167,168].

In addition to the small RNA pathways, the major insect immune signaling pathways, *i.e.*, Toll, IMD and JAK/STAT pathways, also control virus replication in infected mosquitoes, even if the precise mechanisms are still poorly understood [30,129]. These canonical innate immune pathways have been well described and the components and activity of these pathways against different microorganisms have been largely elucidated in *D. melanogaster* and extrapolated to vector mosquitoes as previously reviewed [169]. The major components of these pathways have also been identified in aedine and anopheline vector species [170,171]. As in the fruit fly, these pathways play a role in mosquito immune defences against parasites, fungi, bacteria and viruses [30,129,172,173]. The Toll pathway is activated in the presence of Gram positive bacteria, viruses and fungi as well as the rodent malaria parasite (*Plasmodium berghei*) while the IMD pathway is mainly activated by Gram negative bacteria, viruses and by the human malaria parasite (*Plasmodium falciparum*). The JAK/STAT signaling pathway has been implicated in antibacterial, antiviral, and antiplasmodial defence in mosquitoes. Stimulation of the Toll, IMD and JAK/STAT pathways leads to the activation and nuclear translocation of the transcription factors Rel1, Rel2 and STAT, respectively. This results in the expression of genes encoding antimicrobial peptides (AMPs) and other immune effector genes, leading to pathogen killing [30,129,172,173].

In contrast to the small RNA pathways, the contribution of each immune pathway can be different according to the arbovirus family and to arbovirus–mosquito interactions as described below. In response to DENV, both the Toll and JAK/STAT signaling pathways are activated in *Ae. aegypti*. Moreover, silencing of a negative regulator of the Toll pathway, Cactus, in *Ae. aegypti* mosquitoes results in a decrease in DENV infection of the midgut. Conversely, silencing of Myd88, a component of the Toll pathway, resulted in an increase in DENV titers [115]. Similarly, silencing of the negative regulator of the JAK/STAT pathway, PIAS, leads to a reduction in DENV titers in *Ae. aegypti* mosquitoes, whereas silencing of positive regulators, Hop, Dome, or the JAK/STAT-regulated dengue virus restriction factor 1 (DVRF1) resulted in increased viral loads [41,112]. In the salivary glands of dengue-infected *Ae. aegypti*, both Toll and IMD were found to be up-regulated suggesting that these pathways might control virus infection in this tissue [174,175]. Moreover, a cecropin-like peptide, which has anti-DENV and anti-CHIKV activity, is induced in DENV infected *Ae. aegypti* salivary glands [174]. Thus, the *Ae. aegypti* mosquito vector seems to mount tissue-specific antiviral responses against DENV infection. The combination of the Toll, JAK/STAT and exogenous siRNA pathways appears to control DENV infection at least in the midgut; with Toll and IMD potentially acting as antiviral pathways in the salivary glands. Tissue-specific aspects of antiviral defences are further supported by differences in the overall transcriptome regulation upon DENV infection in different *Ae. aegypti* tissues, *i.e.*, the midgut, salivary glands and carcass [115,174,175]. Interestingly, there are also differing degrees of immune pathway contribution to control virus infection in refractory or susceptible strains of *Ae. aegypti* [44]. For example, the IMD pathway controls DENV infection in the midgut of *Ae. aegypti* resistant strains only, as shown by increased viral loads in IMD depleted mosquitoes. However, the situation is less clear in susceptible strains. Up-regulating the IMD pathway by silencing Caspar, a negative regulator of the IMD pathway, has no effect on midgut DENV infection. However, as IMD has not been directly knocked down in these susceptible strains, it is difficult to tell whether this pathway is already operating at maximum capacity without any endogenous inhibition by Caspar, or whether it is not active or not protective against DENV midgut infection in susceptible strains [44,115]. In addition, DENV-refractory *Ae. aegypti* mosquito strains present higher basal levels of numerous immunity-related gene transcripts compared to susceptible strains suggesting that variation in the transcriptome influences vector competence [44]. Exposure to gut microbiota, which has been shown to stimulate basal antiviral immunity and decrease *Ae. aegypti* vector competence for arboviruses, may explain these transcriptome variations to some extent but inherent differences in gene regulation may also underlie these observations [115].

In the case of *Aedes* mosquitoes infected by alphaviruses, an antiviral function of the canonical innate immune pathways has not been yet demonstrated. However, little evidence supports the hypothesis that they could be involved in *Aedes* responses against this virus family as well. Upon SINV infection of female *Ae. aegypti* mosquitoes, Dif, a Toll pathway-activated transcription factor is increased after one day post-infection in the midgut and malpighian tubules, although it returns to basal level by day 4 post-infection. The authors suggested that this is linked to suppression of Toll pathway activation by SINV once replication has taken place [176]. Additionally, infection of mosquito cells with SINV resulted in the translocation of the Rel2 protein into the nuclear fraction of *Ae. albopictus* C6/36 cells indicating that infection with this virus may also result in IMD pathway activation [177]. Fragkoudis *et al.* and McFarlane *et al.* used reporter assays to investigate the interaction of the innate immune pathways in mosquito derived cell lines with SFV and CHIKV, respectively. In *Ae. albopictus* derived U4.4 cells, SFV can repress activation of the IMD, JAK/STAT and Toll pathways [178]. Similar results were observed in *Ae. aegypti* derived Aag2 cells using CHIKV replicon constructs where pathway induction was also inhibited through host cell shut-off mechanisms [139]. In addition, prior stimulation of the JAK/STAT and/or IMD pathway (the assay cannot distinguish between them) resulted in a decrease in SFV replication, though CHIKV seemed to be unaffected by the activation of the three main immune pathways [139,178]. The contribution of each of the innate immune pathways in different mosquito body compartments during the stages of an alphavirus infection is better characterized in *An. gambiae* infected by ONNV. Unlike *Aedes* infected with flaviviruses, the exogenous siRNA is not the first line of natural antiviral defence in the midgut, but interplay between the IMD and JAK/STAT pathways, as well as extracellular immune factors, limit virus infection [145]. In contrast, the Toll and exogenous siRNA pathways control viral load once the virus has escaped from the midgut into the systemic compartment [145].

Thus, mosquitoes have naturally developed complex antiviral defence strategies with an arsenal of pathways (certainly the RNAi and major immune pathways discussed above) to fight arboviral infections. Moreover, even if more functional studies are needed to highlight differences in vector responses to infection by different arboviral families, these tissue-specific mosquito antiviral strategies appear to be influenced by arbovirus-mosquito strain combinations and thus, by genome evolution of both the mosquito vector and the virus. This can partly explain natural variations in mosquito vector competence for arboviruses which is affected by the specific combination of mosquito and virus genotypes, as well as by environmental factors, such as the naturally present gut microbiota [26,30]. Therefore, copying natural antiviral strategies and engineering mosquitoes with enhanced antiviral defences is a promising approach to decrease mosquito vector competence for arboviruses and to control their transmission.

#### 3.1.2. Limits and Technical Challenges to Engineer Mosquitoes with Enhanced Antiviral Defences

In order to design efficient transgenic mosquitoes with enhanced specific antiviral defences, more experimental research is needed to better understand the contribution of tissue-specific antiviral strategies in the mosquito, defence pathway crosstalk, as well as communication between different mosquito tissues during a viral infection which leads to an integrated antiviral response. For this to be achieved, and to test the influence of different potential targets for each mosquito–virus pair, mosquito genetic tools are essential. *Ae. aegypti* transgenic lines modified to overexpress or to repress the Toll and IMD immune pathways, as well as AMPs (e.g., cecropin and defensin), have already been engineered to decipher the mosquito immune response during mosquito–pathogen interactions; although, these have not yet been tested against arboviruses [179,180,181,182,183,184]. In these transgenic mosquitoes, the immune modifications are driven by the *Vitellogenin* gene (*Vg*) promoter which is responsive to blood feeding and mainly drives expression in the fat body between 12 and 48 h post blood meal [115,181,183,185]. Thus, it may not be the best promoter to assess an infection in the fat body which may occur later during DENV infections. However, these lines could be tested against CHIKV which disseminates from the gut to peripheral organs, such as salivary glands, more rapidly than DENV [186,187]. Moreover, the fat body is a tissue with important immune functions [172], and even if the virus is not yet present or does not disseminate to the fat body, this tissue could participate in the integrated antiviral immune response following communications between tissues triggered by viral midgut infection.

It is necessary to identify mosquito regulatory sequences to drive expression of transgenes in a tissue-specific manner; not only to decipher the molecular basis of mosquito antiviral defences at the tissue-level, but also because in the context of population replacement, a transgene should be limited to a specific time and tissue to achieve the maximum effect on the pathogen with the least fitness costs to the vector [31,188,189]. The choice and design of the promoters should not only take into consideration the tissues with immune properties but also the mechanisms by which arboviruses disseminate in the mosquito, their tissue/cell-specific tropism, and persistence in tissues. From the midgut to the salivary glands, various mosquito organs and cells have been shown to be infected with arboviruses such as the trachea, the fat body, the muscles, the cardia and the head [186,190,191,192,193,194]. Interestingly, the hemocytes (the immune cells of insects) can be infected by arboviruses in different species of *Aedes*, in *Cx. pipiens* as well as in *An. gambiae* [145,195]. Since hemocytes play a role in mosquito immunity [172], it would be interesting to determine if these cells are a site of viral amplification and whether they act as viral transporters in or through the hemolymph and/or if they play a defensive role against viruses. Like the *Vg* regulatory region driving gene transcription in the fat body, different promoter expression patterns have already been characterized and used to drive tissue-specific transgene expression in adult *Ae. aegypti* transgenic mosquitoes. In the midgut, the aedine *Carboxypeptidase A* promoter drives transgene expression in the female midgut after blood feeding [140,166,196,197,198]. The aedine *30K* gene family promoter directs specific expression in the salivary glands of transgenic *Ae. aegypti* females [168]. The *Maltase-like I* gene promoter also drives gene expression in salivary glands, but in both sexes, and the *Apyrase* gene regulatory sequence controls expression in the female salivary glands but also in other tissues [199]. In flight muscles, transgene expression can be directed by the *Actin-4* promoter [200]. Lastly, the regulatory sequence of *Polyubiquitin* (*PUb*) gene drives expression in all stages of development and most tissues. Nevertheless, this promoter drives strong expression in the midgut and to a lesser extent in secondary tissues like the salivary glands [141,201]. It would be useful to identify and characterize more regulatory sequences particularly for the hemocytes or the trachea, for which no regulatory regions have been identified so far. To find candidate promoters for hemocytes, the transcriptomic analysis by Choi *et al.* which compares gene expression between hemocytes and carcasses in *Ae. aegypti* could be used to select some interesting genes up-regulated in hemocytes [202]. Moreover, this study shows that some hemocyte-specific transcripts in *Ae. aegypti* are conserved in *An. gambiae* and *D. melanogaster*, allowing for the selection of aedine promoters that have already been shown to be functional in *D. melanogaster* hemocytes. For the trachea, different gene promoters have been characterized in *D. melanogaster*, such as *breathless* [203], which could be further tested in mosquitoes.

**Figure 2:**
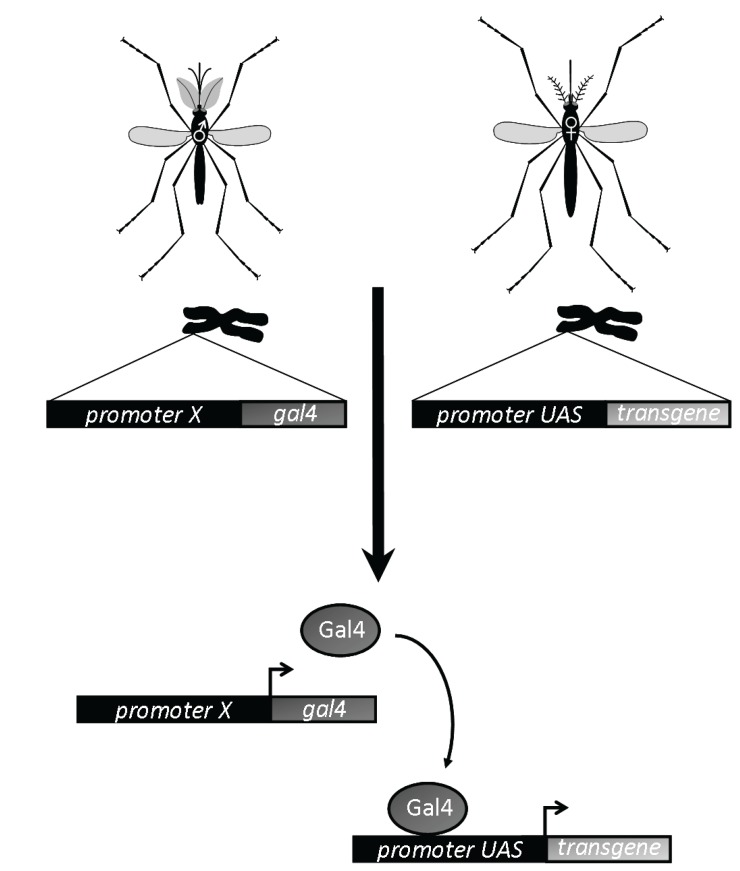
Schematic representation of the Gal4-UAS system. In a first line, the driver line, the yeast transcriptional activation factor Gal4 is under the control of a promoter directing the expression in the tissues/cells of interest. In the other line, the responder line, the transgene of interest is expressed under the control of the upstream activation sequence (UAS). As transcription of the transgene requires the presence of Gal4, the transgene is silent in the parental responder line. When the driver line and the responder lines are crossed, the transgene is then expressed according to the Gal4 pattern.

The Gal4-UAS system, widely used in *Drosophila*, has proven to be one of the most powerful techniques for addressing gene function *in vivo* and for the characterization of regulatory regions [204,205]. A key advantage of the system is the separation of the two components in transgenic parental lines, the Gal4 driver line and the UAS responder line, which ensure that the transgene is silent until Gal4 is introduced through a genetic cross (Figure 2). The Gal4-UAS system is functional in *Ae. aegypti* and two Gal4 lines, *CpA-Gal4* and *Vg-Gal4*, as well as a UAS-EGFP line have been already established [185,198]. The development of this system in aedine mosquitoes, along with advances in transgenic technologies to edit the mosquito genome, such as the site-specific insertion system PhiC31 [206,207,208] or gene knock outs with TALE nucleases [209,210], should facilitate the generation of genetic tools to decipher mosquito antiviral strategies and to engineer refractory mosquitoes. Moreover, the CRISPR/Cas9 editing system, which promises to transform genome engineering in different species including insects [211,212], has been recently adapted in *Ae. aegypti* and allows the generation of mutations as well as homologous recombination [213].

Importantly, mosquito responses to infection can vary depending on the specific arboviruses involved as highlighted above, and more generally among other pathogens too. Thus, decreasing vector competence for one arbovirus could increase vector competence for another pathogen. Besides, a mosquito vector for a given arbovirus could bite people infected with other arboviruses or pathogens in a same geographic area. To avoid shifts in vector competence which could consequently alter their capacity for other pathogens and lead to an increase in non-target diseases, every transgenic mosquito with enhanced antiviral defences for a given arbovirus should be tested for its ability to be infected and transmit other pathogens before any release program is implemented. Immune evasion of arboviruses and their potential evolution should also be considered when designing transgenic mosquitoes with modified defence properties. Indeed, arboviruses may develop counter defences to evade or suppress host defences and persist in the defending mosquito, therefore increasing mosquito competence and virus transmission. The mechanisms are not yet well characterized but may involve, for instance, transcriptional down-regulation of immune genes, decreases in RNAi pathway function as a result of viral suppressors of RNAi, genome nucleic acid sequence- or genome secondary structure-mediated resistance to RNAi [129]. Indeed, almost all arboviruses are RNA viruses, and as such they rapidly replicate with high mutation rates promoting the emergence of new variants. This can therefore lead to an increased fitness in their mosquito hosts as reported for DENV or CHIKV [142,214,215,216,217].

#### 3.1.3. Other Strategies for Engineering Mosquitoes with an Impaired Arboviral Competence

As discussed above, enhancing defence pathways to decrease mosquito competence for a given arbovirus could not be functional for every example. Perhaps, a more viable strategy rather than constitutively activating a defence pathway would be to identify a broadly acting antiviral molecule which could either be overexpressed or knocked down. There have been a number of these types of screens performed in recent years with the aim of finding broad acting antiviral or pro-viral mosquito proteins which have the potential to influence mosquito competence for arboviruses. For example, a comparative transcriptomic analysis of *Ae. aegypti* mosquitoes infected with the flaviviruses DENV, WNV and YFV, identified a number of proteins, including the histone core H3 protein, which are activated or down-regulated in response to infection, suggesting broad pro- or anti-viral roles for these proteins [218]. However, the antiviral role of these proteins in mosquitoes would have to be functionally characterized for these viruses as well as for other arbovirus families before targeting could be considered. Interestingly, this screen identified a reduction in chitin-binding related transcripts upon infection of *Ae. aegypti* with all three flaviviruses and these proteins were also identified in a screen of mosquitoes infected with SINV [176,218]. Similarly, candidate genes responding to DENV infection or those which where differently regulated between refractory and susceptible *Ae. aegypti* strains in the midgut or salivary glands, have been identified again through transcriptomic analyses [44,115,174,175]. For instance, the vacuolar ATPase complex, which regulates endomsomal pH, has recently been identified in one of these screens and was shown to be required for DENV replication in *Ae. aegypti* [44]. In addition, a large RNAi screen in *D. melanogaster* identified hits that have been shown to mediate antiviral activity against a number of virus families. For example, components of the Tip60 complex, including RUVBL1, RUVBL2 and TIP60, and a nuclear export factor, XPO1, were not only shown to be antiviral against a whole range of RNA viruses in *Drosophila* S2 cells but also in *Ae. aegypti* Aag2 cells [219]. Interestingly the proteins identified in this screen have been shown to be antiviral in vector mosquito cell lines as well as in mammalian cell lines which suggests that they have essential roles in the virus life cycle and could prove to be an attractive target for antiviral control in vectors [219]. Additionally, in *Cx. pipiens quinquefasciatus* mosquitoes, in addition to *Cx. quinquefasciatus* and *Ae. albopictus* cell lines, a secreted antiviral protein, Vago, was shown to be induced in a Dcr2-, TRAF- and Rel2-dependent manner in response to WNV and DENV infection. After secretion, Vago activated antiviral gene expression *via* the JAK/STAT pathway, thus restricting WNV replication [220,221]. Targeting host proteins with pro-viral functions such as virus receptors or proteins hijacked by the virus to replicate and disseminate is also an alternative strategy to modulate mosquito competence for arboviruses. However, few proteins and mechanisms have been identified and even less characterized in mosquitoes. Nup98, a protein that promotes the recruitment of the elongating form of RNA pol II at antiviral gene loci and other proteins involved in the transcriptional pausing process, thus allowing for rapid activation of antiviral genes upon virus infection, is important in controlling virus infections in *Drosophila* S2 cells and *Ae. aegypti* Aag2 cells [222,223]. A better understanding of the cells and tissues infected by arboviruses should also facilitate functional characterization of other host cell processes and host factors that are involved in the autonomous cell-control of virus replication, such as autophagy and apoptosis of infected cells, as it has not yet been determined if these cellular processes have an antiviral function or facilitate viral dissemination in mosquitoes [30,130].

Another possible genetic control approach to decrease transmission could be “death-on-infection” as reviewed previously [188]. This involves transgenic mosquitoes carrying a lethal effector activated by pathogens leading only to the death of infected mosquitoes. This original approach acts on vector longevity and not on vector competence and is therefore not discussed here. Nevertheless, Carter *et al.* recently developed an elegant strategy by coupling the targeted ribozyme capabilities of the antiviral group I intron with the generation of an apoptosis-inducing transcript. This leads to virus trans-splicing with the pro-apoptosis transcript resulting in virus genome cleavage and cell death upon infection and has been successfully used to suppress DENV infection in *Ae. albopictus* C6/36 cells [224]. Based on natural infection rates of midgut cells and the regenerative capabilities of midgut epithelia, the authors expect that the loss of cells will not have a significant impact on the survival of the transgenic mosquitoes but will only limit vector competence. Moreover, this system targets all DENV serotypes. Therefore, this “cell death-on-infection” is a promising tool to decrease mosquito competence even if it has not yet been demonstrated to work *in vivo*.

A different death-on-infection-derived strategy would be to increase mosquito vector competence so as to render them hypersensitive to arboviral infection leading to mosquito death. In contrast to arbovirus infections in vertebrates, it has been traditionally considered that arboviruses are benign to their mosquito hosts, leading to “persistent” infections. However, a recent meta-analysis showed that arbovirus infections do reduce mosquito survival [225]. Different hypotheses, mainly arising from studies with the malaria mosquito, may explain the loss of mosquito fitness associated with pathogen infection cost, such as tissue- and cell-pathogenicity, resources competition, and the costs of the anti-pathogen defences [226]. As reviewed above, mosquitoes do naturally fight an arboviral infection. Thus, the “persistence” of arbovirus infections in mosquito vectors is only a reflection of mosquito competence, *i.e.*, the mosquito’s ability to become infected by a virus which can survive long enough to reach the salivary glands and be transmitted. This is partly fine-tuned by a balance between the mosquito antiviral defences to control virus replication and survive the infection *versus* the virulence of the virus to be transmitted without killing the vector [31]. Therefore, it might be possible to imbalance these interplays by decreasing mosquito antiviral pathways (*i.e.*, increasing mosquito vector competence) in favor of the virus, resulting in an increase in virus-induced mortality. In *Ae. aegypti*, a few studies have assessed the survival of mosquitoes which present decreased antiviral defences and increased infection titers through silencing of the exogenous siRNA pathway, and results are contrasting. Two studies have shown a decrease in survival rates of mosquitoes infected by a recombinant strain of SINV that expresses FHV-B2, a suppressor of the siRNA pathway [227,228]. In contrast, some other studies reported no evident decrease in survival in mosquitoes infected with DENV or SINV [135,136,140,141]. In the latter case, it has been suggested that the siRNA pathway was not silenced/inhibited enough to increase virus titers above a certain threshold to induce mortality. Therefore, with the aim of developing immuno-deficient mosquitoes to decrease transmission of arboviruses, further studies would be required to better understand the mechanisms involved in the fitness cost of arbovirus infection on the mosquito.

#### 3.1.4. Future Challenges for Mosquito Population Replacement

Whatever the strategy, a prerequisite for release programs of GM mosquitoes is the stability of the introduced transgene within transgenic strains over many generations [31]. First, transgene expression can vary among and within transgenic insect strains due to position effects, *i.e.*, the influence of neighbouring enhancer or repressor elements on the inserted transgene [229,230]. The use of insulators can avoid these effects and enable stable expression of transgenes [191]. Exogenous insulators from *Drosophila* have already been used successfully to stabilize the expression of transgenes in *Anopheles* mosquitoes [231]. Insulators have also been identified and characterized in *Ae. aegypti* [232] although they have not been demonstrated to be functional in transgenic mosquitoes. Generating GM mosquitoes through site-directed integration, such as the PhiC31 site-specific transformation system, in well characterized docking sites could also reduce position effects [233,234]. Furthermore, fitness losses caused by a transgene, or insertion/expression of an exogenous transgene in an organism may lead to silencing of transgene expression. For example, transgenic lines of *Ae. aegypti* which were resistant to DENV2 in the midgut, lost their resistance phenotype after many rearing generations due to a loss of transgene expression [166,235]. Interestingly, the same authors recently showed that outcrossing a newly established line with the same transgene into genetically-diverse strains over many generations can reduce the fitness cost associated with the transgene [167]. This further supports the inbreeding depression phenomenon previously described in mosquitoes [45].

However, the main issue with genetically manipulating the mosquito defence system is the fitness costs this may have on the mosquito vector [31,236,237,238] even if, in the context of infection, the transgene-mediated resistance can confer a fitness advantage [237,239]. One would have to ensure that the fitness of transgenic mosquitoes is not affected by the effect of the transgene or the insertion *per se* as during release experiments such genetically modified vectors would quickly be outcompeted by wild type vectors and any antiviral effect would be lost. Using site-specific transformation systems and fitness-tested docking lines could overcome fitness loads associated with the transgene insertion site [240,241]. On the other hand, only directing transient expression of the transgene in a tissue- and stage-specific manner, as discussed above (Section 3.1.2), or inducing it only upon arbovirus infection using an arbovirus-induced promoter, should limit the negative effects of the antipathogen transgene [31,188,189]. Moreover, associating the gene which results in a refractory organism with a strong gene drive mechanism to spread the transgene into natural mosquito populations should allow mosquitoes with modified antiviral properties to be engineered that could represent viable tools for disease control [29,31,127,128,188,242,243]. A recent model-based study has evaluated the efficacy of several genetic strategies to control disease vectors. This work has shown that population replacement with GM refractory mosquitoes is easier to maintain and should lead to a greater long-term reduction in competent vectors compared to population reduction approaches, therefore encouraging antipathogen strategies [244].

Finally, ethical and regulatory considerations impose that each GM product must proceed through a risk assessment process to evaluate the benefits and risks which may require a case-by-case approach. Before implementing the release of GM mosquitoes with altered vector competence, the way in which these mosquitoes could be deployed effectively, efficiently and safely in the field remains to be determined and will require semi-field test experiments. Public acceptance will also be key to implementing genetic control of mosquito competence to decrease disease transmission [14,29,127,245].

### 3.2. Using Paratransgenesis Approaches to Decrease Vector Competence for Arboviruses

Paratransgenesis is an approach where endosymbionts commonly found in insect disease vectors are genetically modified to express anti-pathogen effectors so as to reduce insect vector competence. The main characteristics of paratransgenesis are the relative simplicity with which symbionts can be transformed with reduced fitness costs compared to insect transgenesis, the feasibility of the transformed endosymbiont to spread across an insect population, and the insect-specificity [246].

Several characteristics are important for such endosymbionts to be used as successful expression vectors to control mosquito competence for arboviruses, specifically regarding practicality and safety regulations. Firstly, it should be mosquito-specific or at least insect-specific and be unable to infect vertebrates. Secondly, it should have the capability to be stably modified. It would also be useful if it was not pathogenic by itself in the mosquito and had little impact on the host. Its ability to be vertically transmitted could be useful as this would increase the sustainability of the antiviral effect in mosquito populations and reduce the need for additional release experiments. On the other hand, vertical transmission could be a disadvantage due to the limitation of controlling the spread of the modified symbiont.

Although several endosymbionts, such as bacteria or viruses, have these characteristics, the only ones currently being exploited for commercial use in mosquitoes transmitting arboviruses are ISVs, and more particularly mosquito-specific DNVs. The *Ae. aegypti* (*Aae*) DNV was the first reported densovirus isolated from mosquitoes in 1972 [247]. When *Ae. aegypti* larvae are transduced with a recombinant *Aae*DNV carrying a GFP reporter gene, the fluorescent protein is expressed in different mosquito tissues (e.g., gut, malpighian tubules and anal papillae) during the larval and pupal stages [248]. Studies have also been successfully performed in *Ae. albopictus* derived C6/36 cells and larvae using an *Aae*DNV derived vector, to express either an insect-specific toxin [249] or a short hairpin construct for gene specific silencing [250]. The use of DNVs is not limited to *Aedes* species and has also been shown to be efficient in *An. gambiae*. The protein of interest is preferentially expressed in adult mosquitoes in a wide range of tissues, including the fat body and ovaries; however, no expression was detected in the key tissues during arboviral infections, *i.e.*, the midgut and salivary glands [251,252,253]. A disadvantage of DNVs is their small genome, which may not tolerate long additional sequences; therefore, studies so far have used a DNV-based expression system that is dependent on helper plasmids and co-infections with wild type DNV to induce the spread of the recombinant DNV construct [249,250,251]. Arboviruses from different virus families (*Togaviridae*, *Bunyaviridae* and *Flaviviridae*) have been shown to successfully express foreign genes; however, these experiments with their ISV counterparts have yet to be performed. It can be expected that in the future, work performed with DNVs will be broadened to ISVs belonging to other virus families, using the knowledge retrieved from years of arbovirus research.

In addition to ISVs, mosquito bacterial symbionts could also be transformed to express molecules which interfere with arboviral transmission. This is already in development for different insect disease vectors, such as malaria mosquitoes, by using bacteria of the *Asaia* genus [246,254]. Since *Asaia* have been reported to be associated with *Ae. aegypti* and *Ae. albopictus*, using these bacteria to express antiviral molecules might be another paratransgenic strategy to modify mosquito vector competence for arboviruses and decrease mosquito-borne viral disease transmission [254].

In the context of controlling mosquito arboviral vector competence, using genetically modified bacteria or ISVs, efficient arbovirus-specific antiviral molecules still need to be characterized. Moreover, mosquito microbial ecology and the role of this microbiota need further investigations in order to select transformable endosymbionts for each vector. Furthermore, how engineered symbionts can be spread in the field and replace non-transformed symbionts in natural mosquito populations needs to be assessed in both laboratory and field experiments. Additionally, a drive system, such as co-infection with the engineered symbiont and *Wolbachia*, could increase the frequency in mosquito populations of the transformed symbiont, and potentiate the effectiveness of replacement to achieve successful paratransgenic control of mosquito competence for pathogens [246,255].

## 4. Conclusions

**Figure 3:**
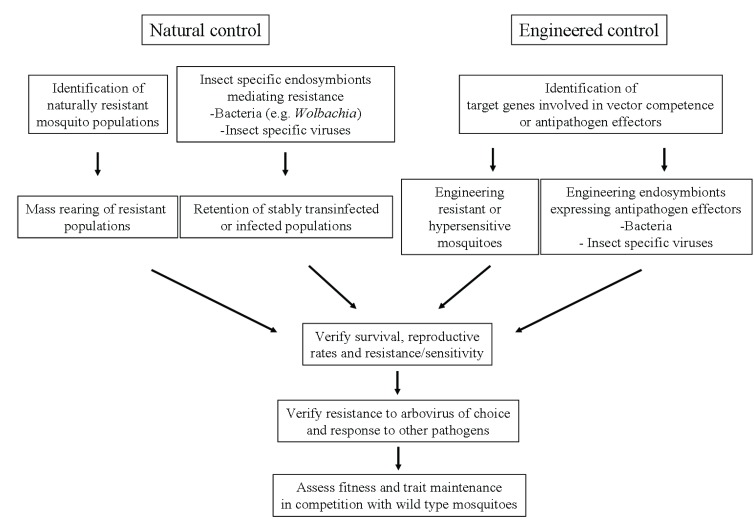
Summary of the currently proposed natural and engineered strategies to decrease mosquito competence for arboviruses.

Here, we summarized the control strategies that are being developed to limit mosquito vector competence (Figure 3). The identification of antiviral pathways in mosquitoes has opened new intervention possibilities; but other factors, such as receptors or any pro-viral protein, are also of interest as targets for modification. Clearly, more information about natural resistance of vectors to arboviruses and the underlying genetic factors is also required. This can inform strategies based on the selection of traits or genetic modification strategies and a number of difficulties are likely to arise. It is not known at present, and it is also difficult to predict, how such genetically modified mosquitoes may fare against their wild type counterparts but this needs to be assessed for each population individually, in particular with regards to the key arboviruses in the study or release area. Paratransgenesis approaches combine several ideas but this field is still novel and the ideal targets still need to be identified. Nonetheless, the use of *Wolbachia* and RIDL technology suggests that vector-based control strategies and our increasing understanding of vector biology are ready to move the field further and improve current measures in vector control. Whatever the strategy to reduce vector competence of *Aedes* spp*.* vectors, a greater understanding of the interactions between arbovirus, mosquito and endosymbionts (such as gut bacteria and ISVs) is needed to avoid any negative impacts, such as the increase in transmission of a non-targeted arbovirus. For the same reason, every strategy will have to be assessed for mosquito vector competence to different mosquito-borne pathogens. In addition, as different mosquito species in a same geographic area can be responsible for arbovirus transmissions, it may be necessary to target more than one mosquito species to efficiently decrease disease transmission. Last but not least, engagement with the public is a critical point in taking such approaches forward and the experience and processes currently being built up will help future efforts.
